# Enhanced genetic algorithm optimization model for a single reservoir operation based on hydropower generation: case study of Mosul reservoir, northern Iraq

**DOI:** 10.1186/s40064-016-2372-5

**Published:** 2016-06-21

**Authors:** Yousif H. Al-Aqeeli, T. S. Lee, S. Abd Aziz

**Affiliations:** Faculty of Engineering, Universiti Putra Malaysia, 43400 UPM Serdang, Selangor Malaysia; Department of Dams and Water Resources Engineering, Faculty of Engineering, Mosul University, Mosul, Iraq; Department of Biological and Agriculture Engineering, Faculty of Engineering, Universiti Putra Malaysia, 43400 UPM Serdang, Selangor Malaysia

**Keywords:** Genetic algorithm, Optimal operation, Hydropower generation, Single reservoir, Mosul reservoir, Iraq

## Abstract

Achievement of the optimal hydropower generation from operation of water reservoirs, is a complex problems. The purpose of this study was to formulate and improve an approach of a genetic algorithm optimization model (GAOM) in order to increase the maximization of annual hydropower generation for a single reservoir. For this purpose, two simulation algorithms were drafted and applied independently in that GAOM during 20 scenarios (years) for operation of Mosul reservoir, northern Iraq. The first algorithm was based on the traditional simulation of reservoir operation, whilst the second algorithm (Salg) enhanced the GAOM by changing the population values of GA through a new simulation process of reservoir operation. The performances of these two algorithms were evaluated through the comparison of their optimal values of annual hydropower generation during the 20 scenarios of operating. The GAOM achieved an increase in hydropower generation in 17 scenarios using these two algorithms, with the Salg being superior in all scenarios. All of these were done prior adding the evaporation (Ev) and precipitation (Pr) to the water balance equation. Next, the GAOM using the Salg was applied by taking into consideration the volumes of these two parameters. In this case, the optimal values obtained from the GAOM were compared, firstly with their counterpart that found using the same algorithm without taking into consideration of Ev and Pr, secondly with the observed values. The first comparison showed that the optimal values obtained in this case decreased in all scenarios, whilst maintaining the good results compared with the observed in the second comparison. The results proved the effectiveness of the Salg in increasing the hydropower generation through the enhanced approach of the GAOM. In addition, the results indicated to the importance of taking into account the Ev and Pr in the modelling of reservoirs operation.

## Background

Many problems in the real world need optimal parameters, which are difficult to find using traditional methods, although they can be found easily by GA (Sumathi and Paneerselvam 2010). The GA is one of the optimization methods that are frequently used to determine the optimal operating policy of water storage systems to obtain the greatest benefit from its management. With regard to these systems, what operators need to know includes knowledge of how much water should be released from the reservoir and when. Every reservoir is designed to meet different downstream requirements relating to water supplies, hydropower, the environment, recreation, and flood control. These demands need to be met in the most effective and reliable way. According to Wurbs (2005), the greatest benefits of storage systems include the maximization of hydropower generation, the maximization of the advantages of irrigation projects based on reservoir water, the reduction of flood damage, and so on. These benefits are represented by objective functions, which have values that change with changes in decision variables. Many studies have been conducted using the GA to derive optimal policies for achieving various objectives at different reservoirs. Wardlaw and Sharif (1999) used a four-reservoir, deterministic, finite-horizon problem to evaluate several alternative formulations of a GA for reservoir systems. Real-value coding, tournament selection, uniform crossover, and modified uniform mutation were chosen for application to find the optimal. A GA approach was applied by Sharif and Wardlaw (2000) to find the optimal solution for a reservoir system in Indonesia. The GA approach was compared with discrete differential dynamic programming (DDDP). GAs with artificial intelligence characteristics were applied to the multi-reservoir system in the Chou-Shui River Basin in central Taiwan (Kuo et al. 2003) to obtain the optimal rule curves for maximizing the benefits for power generation, water supply, irrigation, and recreational purposes. The operational model coupled with use of simulation and GAs for hydropower purposes in multi-reservoir systems was presented by Lin et al. (2005). For case study, the joint operation of the Shihmen and Festui reservoirs in northern Taiwan was chosen. To derive the optimal operational strategies for the Pechiparai Reservoir in Tamil Nadu, India, Jothiprakash and Shanthi (2006) developed and applied a GA model. The objective function was to minimize the annual sum of the squared deviation to obtain the desired irrigation release and the desired storage volume. Reddy and Kumar (2006) derived an optimal operation policy by employing a multi-objective genetic algorithm (MOGA) for a multipurpose reservoir system, the Bhadra Reservoir system, in India. This reservoir serves hydropower generation, multiple purposes irrigation, and downstream water quality requirements. The niche genetic algorithm (NGA) was suggested by Li and Mei (2007) for improving the capability of the traditional algorithm in the optimal operation of reservoir releases. This method has been adopted to derive the optimal operation policy for the cascaded hydropower stations of the Qing River, China. Azamathulla et al. (2008) compared the performance of a GA and the linear programming of by applying them to real-time reservoir operation in an existing Chiller reservoir system in Madhya Pradesh, India. These models were designed to maximize reservoir operation by using knowledge of the total irrigation demand. Hinçal et al. (2011) explored the efficiency and effectiveness of the GA in the optimization of multi-reservoir systems. This model was used to maximize the energy production of three reservoirs in the Colorado River Storage Project in the USA. Xu et al. (2012) suggested a new form for the GA in the operation of a multi-reservoir system consisting of five reservoirs. The performance of this proposed GA was evaluated by comparing its results with those of the traditional GA. Based on a multi-use reservoir system, the optimal rule curves were derived by Ngoc et al. (2014) for Dau Tieng Reservoir, located in the upper Saigon River in southern Vietnam. A penalty strategy and a constrained genetic algorithm (CGA) were applied. This model was simulated for 240 months and evaluated through a generalized shortage index (GSI).

In this study, the GAOM was formulated to maximize the annual hydropower generation for a single reservoir by proposing a new simulation approach to the reservoir operation. In order to achieve this aim, two algorithms were formulated in the GAOM independently. The first algorithm (Falg) is built according to the simulation process of reservoir operation, while the Salg improves the performance of GAOM by enhanced the simulation approach of reservoir operation. In the Falg, the population values of GA (releases to the powerhouse) that were initialized or obtained through GA operations are unchanged, whilst those releases are changed by using the Salg according to some constraints in order to enhance the GAOM. Historical monthly data of Mosul reservoir were used in the GAOM to evaluate those two algorithm for 20 scenarios (years), from October 1989 to December 2009. The optimal annual hydropower produced using these two algorithms were compared with the actual hydropower. All of this was done by running the GAOM in each year separately prior to the inclusion of Ev and Pr in the water balance equation. In the next step, the volumes of Ev and Pr were taken into consideration by using the Salg that strengthened the performance of GAOM. By using this procedure, the GAOM determined the optimal annual hydropower generation during the same 20 years mentioned previously. In this case, each optimal value was compared with its counterpart, which was identified without taking into consideration the Ev and Pr. In addition, it was compared with the observed hydropower. In this work all computations were performed using a Dell Inspiron 14R laptop with an Intel (R) Core i7-4500U CPU @ 1.80 GHz 1.80 GHz, installed memory (RAM) of 8.00 GB, Windows 7 (64-bit) operating system, and Matlab 2013b environment.

## Methodology definition

### Framework

The relationships among the components of a water storage system are generally relatively complex. These relationships express the dynamics of that system through the continuity equation and other physical constraints. The operators of the water storage system usually seek to achieve the highest benefits from operating the system, with a commitment to implement all of those constraints. These benefits are expressed by the objective function. The objective function of this study is maximization of annual hydropower generation, while the constraints include water balance equation, and constraints of storage and release. The constraints of storage include the limits of the calculated storage at the end of each time period, and the limits of storage at the end of the last month of the year. The constraints of releases include the limits of releases to hydroelectric station, and the limits of the total releases from the reservoir. The objective function and the constraints that mentioned above were used in the built of the GAOM to improve the optimal operation policy for a single reservoir by formulating two algorithms. The Falg is based on the traditional simulation of reservoir operation, whilst the Salg has enhanced the work of GAOM through changing the population values of GA according to some constraints if achieved. These population values represent the releases to the powerhouse, which initialized or obtained through GA operations.

Generally, GA looks for the optimal solution through several processes such as selection, crossover, and mutation. These processes represent the basic elements of GA. Each of these elements has many forms which change according to the nature of the problem to be solved. Therefore, these forms of processes should be selected to be appropriate for solving the problem and finding the optimal solution (Roeva et al. 2013). The form of these elements can be selected by relying on previous studies. The work of Hinçal et al. (2011) pertaining to investigate the parameters of GA that used in an optimization problem of water storage system, was used in this study. These parameters include encoding, representation function, selection function, crossover function and probability of mutation. In present study, fitness function referred by Back et al. (1997) was used. The number of generations and population size were determined in the two algorithms formulated independently, in a manner similar to the method used by Roeva et al. (2013).

This GAOM was applied and evaluated by using those two algorithms independently in 20 scenarios for operation of single reservoir in terms of annual hydropower production. This evaluation was done prior to the inclusion of Ev and Pr to the continuity equation. From this evaluation, the best algorithm was identified. Secondly, in order to determine the effect of Ev and Pr on the objective function, the GAOM was evaluated by using the best algorithm in the same scenarios through adding the volumes of Ev and Pr to the continuity equation.

### Objective function

The capacity of hydropower plant basically is a function to water head and flow rate through the turbines. The water head is the difference between the elevations of storage in the reservoir to the tail water depth. Project design concentrate on both of these variables, and on the capacity of hydropower plant. The production of hydropower generation as energy during any period for any reservoir is dependent on several factors: the plant capacity; the flows through the turbines; the average storage head; the number of hours in the operating period; and a constant to convert the flows, water heads and plant efficiency to electrical energy (Loucks et al. 2005).

The objective function of the GAOM in this study was to maximize the annual hydropower generation, as shown below in Eq. (1) (Loucks et al. 2005). This optimal operating policy includes the optimal monthly releases to the hydroelectric station and the monthly levels corresponding to them. In general, these releases should meet the monthly water requirements downstream of the reservoir throughout the year.

1 $$Maximize\,E = \mathop \sum \limits_{t = 1}^{12} k*RP_{t} *H_{t} *Dt/10^{3}$$

*E*: hydropower over 12 months (GWh), *K*: constant (0.003), *RP*_*t*_: release to the hydroelectric station (MCM), *H*_*t*_: average head in the time period (m), *t*: time (month), *Dt*: number of hours in the time period.

The value of the constant k was obtained from the derivation of the hyropower Eq. (1) after assuming that the efficiency of the hydropower station was 80 %. The efficiency range of modern hydroelectric power stations that have been designed properly is generally between 70 % and a maximum of 90 % (Kaltschmitt et al. 2007), so, in this study, the efficiency of the power plant was considered 80 %, which represents the average.

In Eq. (1), the average head during the time period was calculated by subtracting the tail water depth from the average elevation in each month, which depends on the average storage. This average storage was determined from the volumes of storage at the beginning and end of each time period, which were calculated by using the water balance Eq. (2) (Loucks et al. 2005). This equation calculates the storage at the end of the time period, which is taken as the storage at the beginning of the next time period, and so on.

### Constraints

To access the optimal solution, this operation should be subjected to several constraints. These constraints express the behaviour of the water storage system in a realistic way. The first of these constraints is the continuity Eq. (2) for the reservoir.

2 $$S_{t + 1} = S_{t} + I_{t} - R_{t}$$

*S*_*t*+1_: storage at beginning of time period (t + 1), *S*_*t*_: storage at beginning of time period (t), *I*_*t*_: inflow during time period (t), *R*_*t*_: outflow during time period (t), *t*: time period (month).

Note: The units used in Eq. (2) are usually millions of cubic metres (MCM).

Also, the storage should be equal to or below the maximum operational storage and equal to or above the minimum operational storage. That is,

3 $$S_{min} \le S_{t + 1} \le S_{max}$$

*S*_*min*_: minimum operational storage, *S*_*max*_: maximum operational storage.

Release to the hydroelectric station should be between the maximum allowable value, which represents the capacity of these tunnels, and the minimum allowable value, which represents monthly water requirements. These requirements include water for fisheries development, forestry, population, industrial, and thermal energy. Therefore,

4 $$D_{t} \le RP_{t} \le PC$$

*PC*: capacity of tunnels connected to the hydroelectric station, *D*_*t*_: monthly water requirements.

Total releases should be smaller than or equal to the maximum allowable value, which represents the hydraulic capacity downstream of the reservoir. Thus,

5 $$R_{t} \le HC$$

*HC*: hydraulic capacity in the river downstream the reservoir.

The storage at the end of the last month should be equal to the target storage or above. So,

6 $$S_{e12} \ge S_{T}$$

*S*_*e*12_: storage at the end of the last month, *S*_*T*_: target storage at the end of the last month.

A penalty function is used for these constraints, except for the continuity equation, and they are embedded into the objective function. Consequently, the constrained optimization problem takes the form of an unconstrained optimization problem. This procedure is adopted to handle the problem when using a GA.

The constraints of storage are expressed as a penalty function, as follows:

7 $$\left( {S_{t + 1} ,S_{min} } \right) = C_{1} *\left[ {S_{t + 1} - S_{min} } \right]^{2} \quad {\text{For}}\;\;S_{t + 1} < S_{min}$$

8 $$\left( {S_{t + 1} ,S_{max} } \right) = C_{2} *\left[ {S_{t + 1} - S_{max} } \right]^{2} \quad {\text{For}}\;\;S_{t + 1} < S_{max}$$

9 $$\left( {S_{e12} ,S_{T} } \right) = C_{3} *\left[ {S_{e12} - S_{T} } \right]^{2} \quad {\text{For}}\;\;S_{e12} < S_{T}$$

The constraints of the release from outlets leading to the power house and the total release are expressed as a penalty function, as follows:

10 $$\left( {RP_{t} ,D_{t} } \right) = C_{4} *\left[ {RP_{t} - D_{t} } \right]^{2} \quad {\text{For}}\;\;RP_{t} < D_{t}$$

11 $$\left( {RP_{t} ,PC} \right) = C_{5} *\left[ {RP_{t} - PC} \right]^{2} \quad {\text{For}}\;\;RP_{t} > PC$$

12 $$\left( {R_{t} ,HC} \right) = C_{6} *\left[ {R_{t} - HC} \right]^{2} \quad {\text{For}}\;\;RP_{t} > HC$$

$$C_{1} , C_{2} , C_{3} , C_{4} , C_{5} , C_{6} \quad {\text{Constants}}$$

These constants were chosen arbitrarily in order to reduce the possibility of selecting the objective function value when one or more of these penalties become effective.

The deviations from maximum and minimum storage, target storage at the end of the last month, maximum and minimum releases from the tunnel of the hydroelectric station, and total releases are penalized as squares of deviations from constraints.

### The two algorithms formulated

**Fig. 1:**
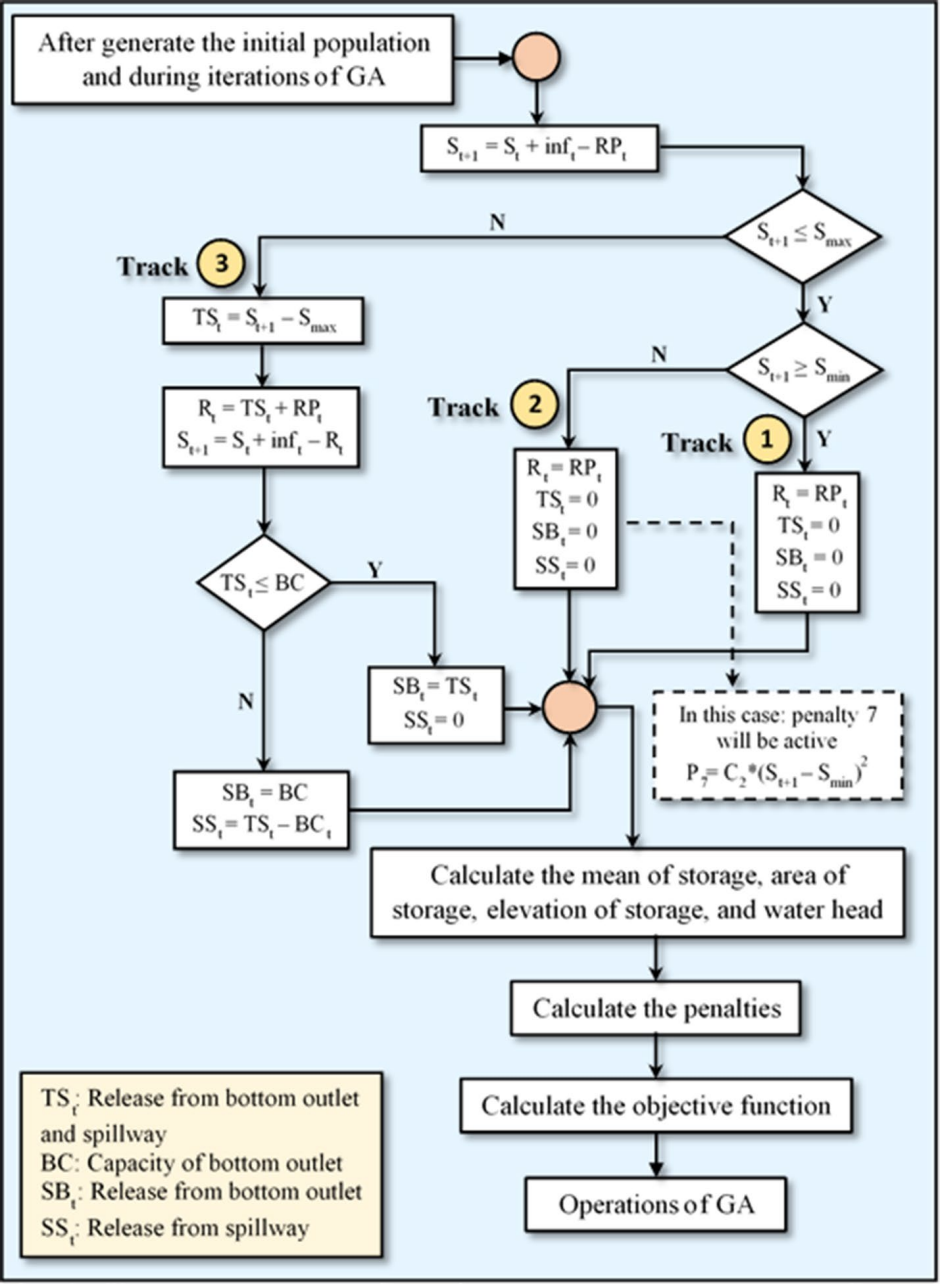
Flowchart of the Falg (traditional GAOM)

**Fig. 2:**
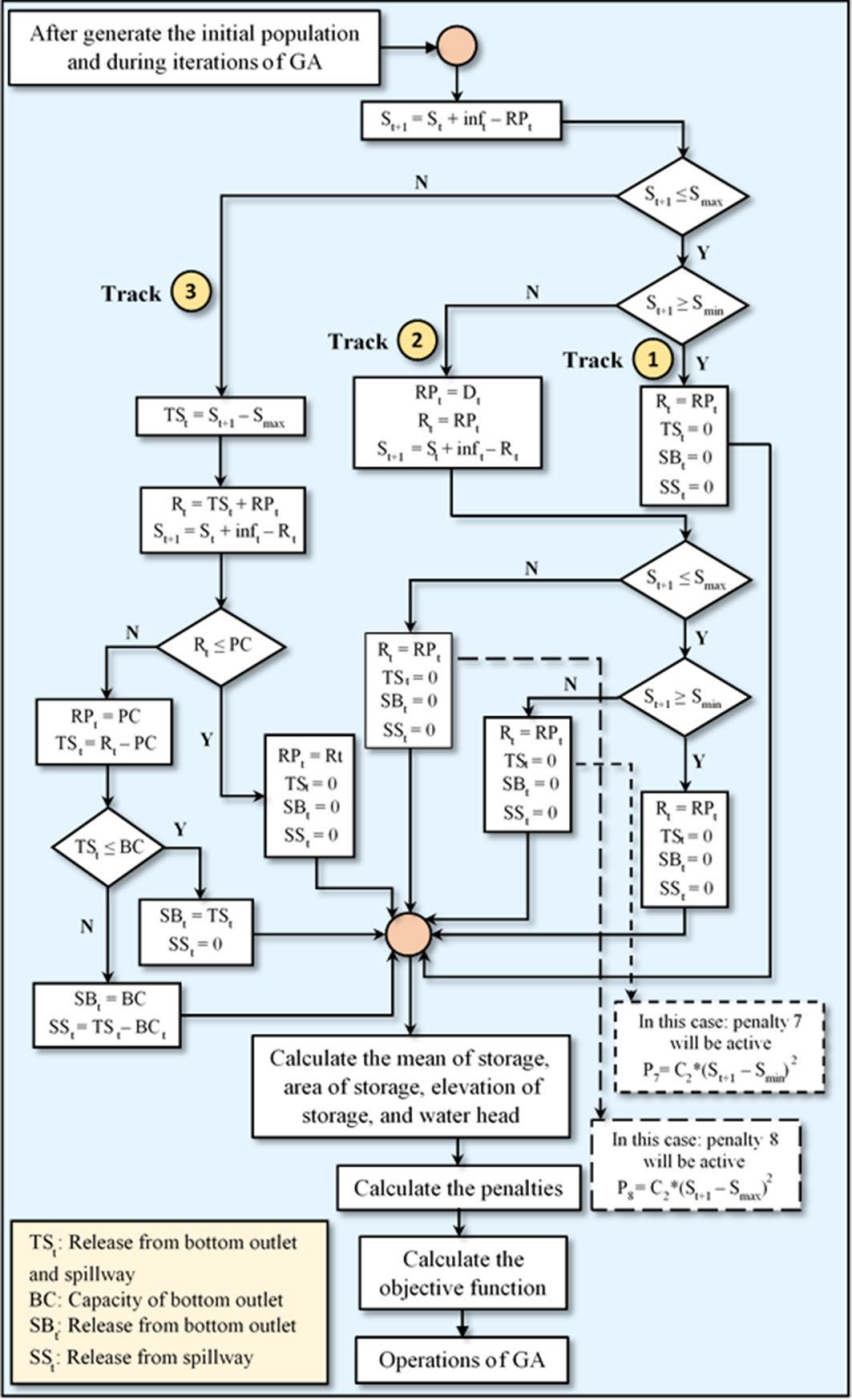
Flowchart of the Salg (enhanced GAOM)

In this study, two algorithms were formulated and used independently in the GAOM. In these two algorithms, firstly, the storage was calculated using the continuity Eq. (2) at the end of each time period depending on the release from the outlets of the powerhouse (*RP*_*t*_) instead of the total release (*R*_*t*_) as shown in the flowcharts of these two algorithm in Figs. 1 and 2. According to the location of this storage in the reservoir, one track is chosen from three tracks as shown in flowcharts of those two algorithm. These tracks represent the zones of storage in the reservoir. These zones of storage are clarified below:

- Zone one: *S*_*min*_ ≤ *S*_*t*+1_ ≤ *S*_*max*_
- Zone two: *S*_*t*+1_ < *S*_*min*_
- Zone three: *S*_*t*+1_ > *S*_*max*_

After that, the *RP*_*t*_ will be replaced by the *R*_*t*_ in the water balance Eq. (2), and the storage will be recalculated. In addition, the average storage for each time period is calculated according to the storage at the beginning and end of each time period. Relying on this average storage, the elevation of storage and water head are determined. This water head is used in the equation of hydropower generation (1). In these two algorithms, the total outflow was separated into three types of releases in each period as shown in their flowcharts. These outflows are released, firstly from the powerhouse outlets, secondly from the bottom outlets, and finally from the spillway, depending on the capacities of these outlets. The working mechanism of each algorithm is explained in the following two sections.

#### The first algorithm (Falg)

Through this algorithm the releases to the powerhouse (*RP*_*t*_) that were initialized or obtained through GA operations are unchanged in all three tracks as shown in Fig. 1. In this algorithm, it should be noted that the penalty function 8 was neglected. This algorithm follows the traditional simulation of reservoir operation.

#### The second algorithm (Salg)

Through this algorithm the releases to the powerhouse (*RP*_*t*_) that were initialized or obtained through GA operations, which represent the population of GA, are changed according to the calculated storage, in the second and third tracks as shown in Fig. 2. These measures are done in order to improve the traditional approach of GAOM in the operation of water reservoirs, where,
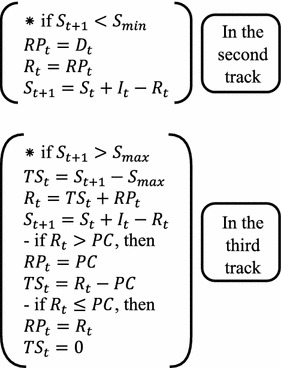
*TS*_*t*_: total release from bottom outlet and spillway.

### Effect of evaporation and precipitation

The effect of Ev and Pr on the values of the objective function depends on their quantities and the differences between them. These quantities and differences relies on the area of storage and the geographic location of the reservoir on the planet. Based on that, the Ev and Pr may have a positive or negative impact on the value of the objective function, or may not have any effect. Relying on the objective function used in present study, the expected effect of these two parameters is positive, if they lead to increase the annual hydropower generation, whilst their impact will be negative, if the opposite happened. To identify the effect of Ev and Pr on the operation of the reservoir, their volumes as measured by MCM were added to the continuity Eq. (2) (first constraint) to become as shown in Eq. (13). The volume of seepage was neglected because, unfortunately, no data are available.

13 $$S_{t + 1} = S_{t} + I_{t} - R_{t} - Ev_{t} + Pr_{t} - Sp_{t}$$

*Ev*_*t*_: evaporation in time period (t), *Pr*_*t*_: precipitation in time period (t), *Sp*_*t*_: seepage from reservoir in time period (t) (neglected).

In each time period, the volumes of precipitation *Pr*_*t*_ and evaporation *Ev*_*t*_ were calculated by multiplying their monthly depth rates by the average of the water surface area. This surface area of water was determined according to the average of storage at the beginning and end in each time interval by using the storage-area curve.

## Case study (Mosul reservoir)

**Fig. 3:**
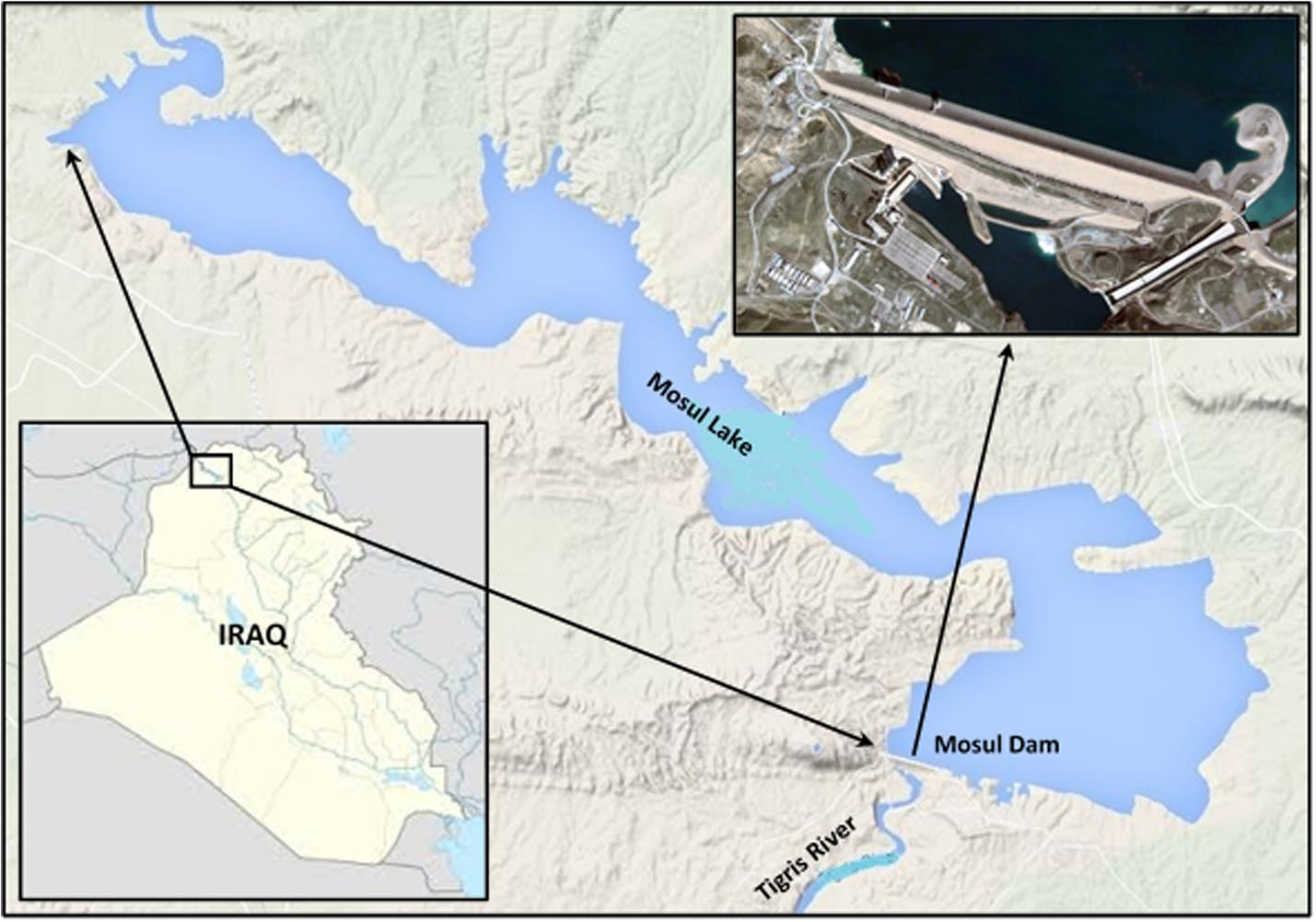
Site of the Mosul dam

**Fig. 4:**
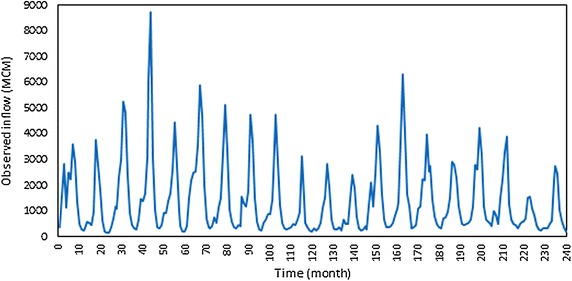
The observed monthly inflow to Mosul reservoir

**Fig. 5:**
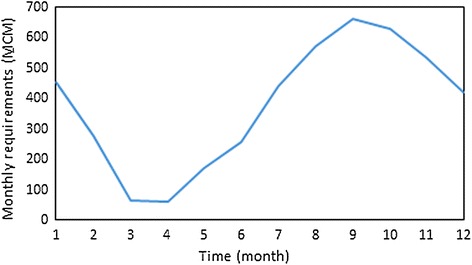
The monthly requirements downstream Mosul reservoir

The Mosul reservoir considered in this study is situated on the Tigris River in Iraq and is about 50 km north of Mosul city (Fig. 3). It is considered Iraq’s largest reservoir. The geographical coordinates in location of the Mosul Dam are 36°37′49″N 42°49′23″E. It began operating in August 1988. The watershed area upstream from the Mosul reservoir covers approximately 50,200 km^2^. The Mosul reservoir serves multiple purposes, including flood control, hydropower generation, and irrigation of large parts of the Al-Jazera region, fisheries, and the development of the tourism sector in the country. The Mosul dam is a rockfill dam with a central clay core. The height of the dam is 113 m, and its length at the top is 3650 m, including 50 m for a spillway. The width of the dam is 10 m at its top and the elevation of its top is 341 m. The spillway has five radial gates for controlling the release of water. The dam has four penstocks/tunnels leading to turbines at the hydroelectric station. The maximum capacity of the hydroelectric station is 772 MW. The bottom outlet is composed of two outlets with segment gates. The tail water depth downstream the reservoir is 265 m. In addition, the elevations and volumes of storage in the Mosul reservoir are shown in Table 1. The values of elevations are taken above mean sea level through some level sensors. The outlets capacity of generating units 1120 m^3^/s, whilst is 1900 m^3^/s for the bottom outlet and 10,000 m^3^/s for spillway. The observed inflows of Mosul reservoir and the demands downstream the reservoir, that used in this study were shown in Figs. 4 and 5 respectively.

**Table 1:** The elevations of storage and the volumes of storage in Mosul reservoir

Minimum level of operational storage (m)	Maximum level of operational storage (m)	Minimum operational storage of reservoir (MCM)	Maximum operational storage of reservoir (MCM)
300	330	2950	11,100

## Determination of elements in the GA

The form of the elements of the GA was chosen by referring to Hinçal et al. (2011), which included verification of a known global optimization problem for water storage system. These elements are representation of the initial population, the selection mechanism, crossover and its probability of occurrence, and the probability of occurrence of mutation. The fitness function that mentioned by Back et al. (1997) was used in the present study. Other elements, such as the population size and number of generations, were identified by operating the GAOM using the continuity Eq. (2) as explained later in this section. All these elements of GA are explained in detail below:

1. Encoding: Real coding was used in the GAOM. According to Michalewicz (1996), and Walters and Smith (1995), the best technique used for the function optimization problem is real-number encoding. It has been extensively confirmed that real-number encoding functions more effectively than grey or binary encoding for optimization problems (Gen and Cheng 2000).
2. Representation function: In order to initialize and represent the initial population in the GAOM, the dynamic coding shown in Eq. (14) was employed. This dynamic coding was proposed by Oyama et al. (2000).
  14 $$x_{i,v} = \left( {x_{v}^{max} - x_{v}^{min} } \right)*rn_{i,v} + x_{v}^{min}$$
  where *i*: Number of chromosomes (individual), *v*: Number of genes (variables) (12), *x*_*i*,*v*_: Initial population (represents *RP*_*t*_), $x_{v}^{max}$: The maximum value of gene (represents *PC*), $x_{v}^{min}$: The minimum value of gene (represents *D*_*t*_), *rn*_*i*,*v*_: Random number located within the range, $0 \le rn_{i*v} \le 1$.
3. Fitness function: As the genetic representation is well-defined, the process continues to the determination of the fitness functions related to the solutions. For a fitness function, the distance between good and bad solutions is calculated by mapping the solution to a non-negative interval. According to Back et al. (1997), there is a mapping that correspond to maximizing problems, as shown in Eq. (15).
  15 $$fitness \left( {\overrightarrow {{va_{i} }} (n)} \right) = 1/\left( {1 + f_{max} \left( {\overrightarrow {{va_{i} }} (n)} \right) - f\left( {\overrightarrow {{va_{i} }} (n)} \right)} \right)$$
  in which *f*_*max*_ represents the maximum observed value of the objective function up to generation (*n*) and *f* represents the objective value.
4. Selection function: Roulette wheel selection was used as a selection mechanism. The basic idea behind this process is to make a random selection from a generation and create a base for the next generation. The fitter individuals have a higher chance of survival than the weaker ones. This process follows nature in the sense that the individuals that tend to have a higher probability of survival will move toward forming the mating pool for the following generation. Obviously, these individuals are likely to have a genetic coding that could prove useful for future generations (Sumathi and Paneerselvam 2010). In this selection, the individuals from the population are considered to be like the slots of the roulette wheel. Each slot is as wide as the probability of selecting the related chromosome. The fitness function, which has already been scaled, is used to compute the corresponding selection probabilities, as shown in Eq. (16) (Shopova and Vaklieva-Bancheva 2006):
  16 $$Pr\left( {\overrightarrow {{va_{i} }} (n)} \right) = fitness \left( {\overrightarrow {{va_{i} }} (n)} \right)/\mathop \sum \limits_{i = 1}^{T} fitness \left( {\overrightarrow {{va_{i} }} (n)} \right)$$
  where *T* represents the population size and *Pr* is the probability.
5. Crossover function: BLX-α was chosen as a crossover function. According to Shopova and Vaklieva-Bancheva (2006), blend crossover has been found to be the most common recombination approach in real representation. Through this scheme, two parents *X*^1^ and *X*^2^ are combined to produce two offspring *Y*^1^ and *Y*^2^, while a new value is sampled. This occurs at $\left[ {\hbox{min} \left( {x_{i}^{1} ,x_{i}^{2} } \right) - \alpha p_{i} ,\hbox{max} \left( {x_{i}^{1} ,x_{i}^{2} } \right) + \alpha p_{i} } \right]$, where each position *i* is defined through Eqs. 17 and 18 (Tomasz 2006):
  17 $$y_{i}^{1} \in \left[ {\hbox{min} \left( {x_{i}^{1} ,x_{i}^{2} } \right) - \alpha p_{i} ,\hbox{max} \left( {x_{i}^{1} ,x_{i}^{2} } \right) + \alpha p_{i} } \right]$$
  18 $$y_{i}^{2} \in \left[ {\hbox{min} \left( {x_{i}^{1} ,x_{i}^{2} } \right) - \alpha p_{i} ,\hbox{max} \left( {x_{i}^{1} ,x_{i}^{2} } \right) + \alpha p_{i} } \right]$$
  where $p_{i} = Abs(x_{i}^{1} - x_{i}^{2}$), and *α* represents a positive real parameter. In the present work, α = 0.1 was used, and the probability of crossover occurrence = 0.7.
6. Mutation function: Uniform mutation was adopted as a mutation function. According to Back et al. (1997), this is a simple mutation scheme. In uniform mutation, the positions of the genes that are likely to mutate are determined at the beginning. The chance of mutation is equal for all genes, but only those for which the mutation probability has taken place will undergo mutation. Then, the new gene is produced that replaces the selected ones. They are randomly selected out in uniform distribution from the search space (0, 1). The uniform mutation operator substitutes the value of the selected gene, which has a uniform random value between the user-determined upper and lower limits for that gene. The only application of this mutation operator is for float and integer genes (Sumathi and Paneerselvam 2010). In this work, the probability of a mutation occurring is equal to 0.02.
7. The population size and number of generations were identified in a manner similar to the method used by Roeva et al. (2013). For this purpose, the GAOM of Mosul reservoir was operated using the two algorithms independently, based on three assumptions. The first assumption: the initial storage was set to equal the average of the minimum and maximum operational storage; the second assumption: the target ending storage of the last month is equal to or greater than the minimum operational storage; the third assumption: The average year of inflow for the Mosul reservoir (monthly rate for 20 years) was used.

The population size and number of generations were identified by operating the GAOM 130 times using the Falg and also 130 times using the Salg, as explained below:

- Operation of the GAOM using the Falg: In Table 2, from cases No.1 to No.6, the number of generations was set equal to 250, and then the population size was changed from 250 to 1500. From these cases, the population was chosen to be 1000, which represents the best population size. In the same table, from cases No. 7 to No. 13, the population size was set equal to 1000, and after that the number of generations was changed from 500 to 6000. From these cases, the number of generations was set equal to 4000, which represents the best value. For each case of population size and number of generations, the GAOM was run ten times to obtain the values of the objective function.

**Table 2:** Determination of the statistical parameters for the values of the objective function obtained relying on the population size and number of generations, using the first algorithm

No. of cases	Population size	No. of generations	Mean	SD	Max	Min	Avr. of Exec. time (min)
1	250	250	2938	21.5	2978	2905	2
2	500	250	2953	17.1	2973	2929	4
3	750	250	2960	10.9	2974	2943	5
*4*	*1000*	*250*	*2966*	*11.3*	*2979*	*2943*	*7*
5	1250	250	2960	12.9	2976	2938	9
6	1500	250	2964	11.4	2977	2941	11
7	1000	500	2963	16.8	2987	2935	14
8	1000	1000	2972	12.6	2992	2956	28
9	1000	2000	2977	11.7	2989	2955	56
10	1000	3000	2983	10.2	2998	2964	84
*11*	*1000*	*4000*	*2984*	*8.2*	*2998*	*2972*	*119*
12	1000	5000	2977	9.0	2993	2965	147
13	1000	6000	2979	8.3	2989	2962	185

The italics indicate to the population size and number of generations which were selected, using the first algorithm
- Operation of the GAOM using the Salg: All of the steps referred to above in relation to the Falg were repeated by using the Salg, as shown in Table 3. From the first six cases, the population size was identified equal to 1000, which represents the best, and from cases No. 7 to No. 13, the number of generations was set equal to 3000, which represents the best value. The GAOM was run ten times in each case of population size and number of generations.

**Table 3:** Determination of the statistical parameters for the values of objective function obtained relying on the population size and number of generations, using the second algorithm

No. of cases	Population size	No. of generations	Mean	SD	Max	Min	Avr. of Exec. time (min)
1	250	250	2962	16.9	2993	2937	2
2	500	250	2971	11.7	2984	2947	4
3	750	250	2974	11.6	2991	2955	5
*4*	*1000*	*250*	*2985*	*8.9*	*3002*	*2971*	*7*
5	1250	250	2979	11.3	3002	2961	9
6	1500	250	2980	10.7	3000	2963	11
7	1000	500	2983	11.3	2994	2958	15
8	1000	1000	2986	10.7	2999	2971	29
9	1000	2000	2993	12.5	3005	2973	57
*10*	*1000*	*3000*	*2996*	*8.4*	*3008*	*2983*	*85*
11	1000	4000	2996	12.6	3007	2971	112
12	1000	5000	2996	10.2	3009	2973	144
13	1000	6000	2999	7.3	3006	2982	173

The italics indicate to the population size and number of generations which were selected, using the second algorithm

**Fig. 6:**
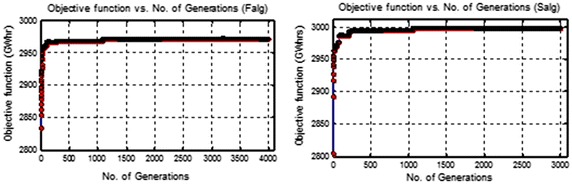
The work progress for the GAOM using the two algorithms

In these tables, the statistical parameters for the values of the objective function were used as functions of the evaluation to identify the best values of population size and number of generations. In addition, these best population size and number of generations which were chosen, represent the optimal choice with respect to the execution time. The work progress of the GAOM using the two algorithms is shown in Fig. 6. This figure shows the hydropower generation obtained using the first and second algorithms, as an example.

## Application and evaluation of the GAOM

### Using the two algorithms without taking into consideration the effect of Ev and Pr

In this study, the objective function of the GAOM was to maximize the annual hydropower generation according to the historical monthly inflows of Mosul reservoir. To evaluate the performance of the GAOM, each of the two algorithms was applied during 20 scenarios in the operation of Mosul reservoir. These scenarios represent the number of years during the chosen period from October 1989 to September 2009, where the water year in Iraq starts on October and ends on September in the following year. That means that the GAOM was run 20 times using each algorithm, giving a total of 40 runs. In each scenario (year), the storage at the beginning of the first month should be inserted in the continuity Eq. (2) in order to operate the GAOM, in addition to the constraint of the target storage at the end of the last month should be known. Based on that, the initial storage (*S*_*t*_) was set to equal the observed storage on the first day of the year, and the calculated storage at the end of the last month (*S*_*e*12_) should equal to or greater than the observed storage on the first day of the following year, which represents the target storage (*S*_*T*_). This constraint was applied in all twenty scenarios (operating years) so as to make the operation of the GAOM subject to the same real operational conditions. In addition, the continuity Eq. (2) and other constraints described previously were applied. The annual hydropower generated when using each algorithm was compared with the observed annual hydropower generated and with that generated when using the other algorithm. The execution times of these two algorithms were also compared. From these comparisons, the better of these two algorithms was identified.

### Using the best algorithm (Salg) while taking into consideration the effect of Ev and Pr

**Fig. 7:**
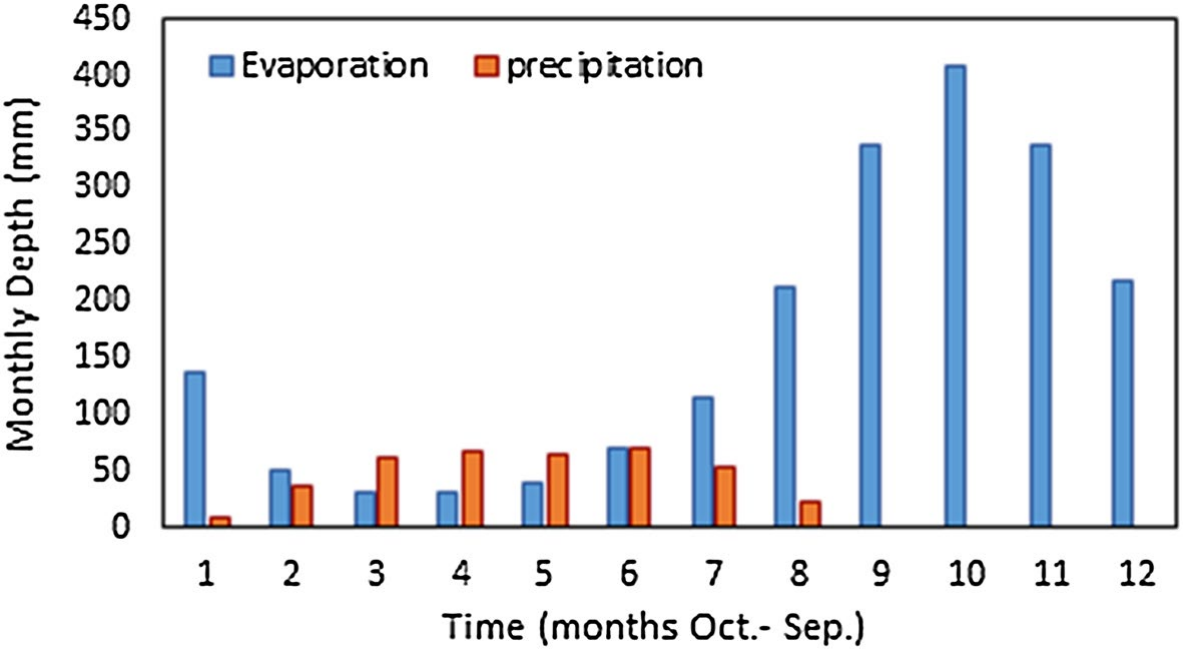
The monthly evaporation and precipitation rates of Mosul reservoir

In order to evaluate the performance of the GAOM using the Salg, with taking into consideration the effect of Ev and Pr, the volumes of these parameter were added to the continuity equation as shown in Eq. (13). In this study, the monthly Ev and Pr rates of Mosul reservoir, that shown in Fig. 7 were used. The GAOM was applied using the Salg, which represents the best, during the same 20 scenarios that mentioned previously. The values of the objective function were compared twice, first with those resulting from use of the Salg while ignoring the Ev and Pr and second with the observed hydropower generation. From these comparisons, the effect of Ev and Pr was identified.

## Results and discussion

As stated previously, the optimization model was applied in three phases as explained below:

In the first phase, the GAOM was applied to determine the population size and number of generations for the two algorithms. The population size and number of generations were set as 1000 and 4000 for the Falg, whilst they were set equal to 1000 and 3000 for the Salg. These population size and number of generations which were chosen, represent the optimal choice with respect to the statistical parameters obtained and the execution time.

**Fig. 8:**
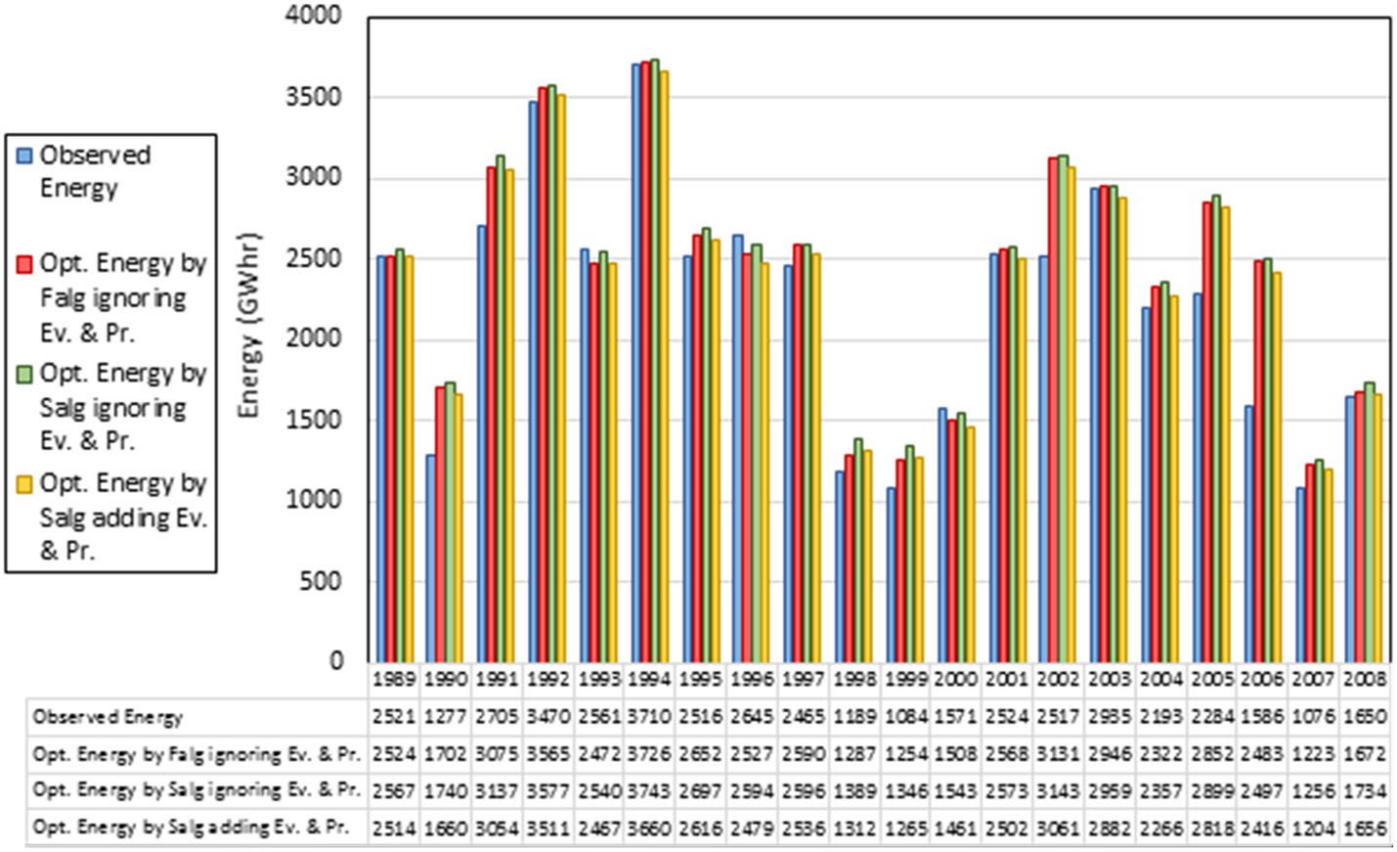
The observed values and the values of the objective function using the two algorithms

**Fig. 9:**
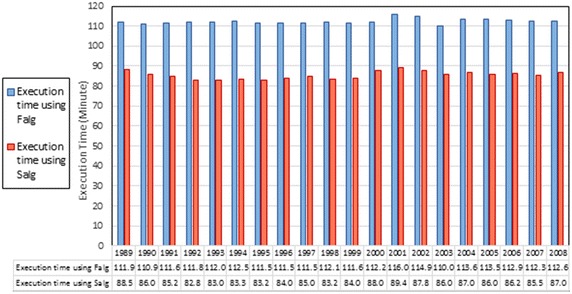
The execution time of the GAOM using the first and second algorithms

In the second phase, the GAOM was applied without taking into consideration the volumes of Ev and Pr, using two algorithms independently. Each of these two algorithms was applied during 20 scenarios in the operation of Mosul reservoir. In this phase, the values of the objective function obtained by using the first and second algorithms were compared, and with the observed values as shown in Fig. 8. As well, the increments and decrements in the hydropower generation achieved as percentages by using those two algorithms were compared as shown in Table 4. These comparisons indicated that the optimal values of annual hydropower generation using the two algorithms were often better than observed during all 20 scenarios used. These two algorithms achieved an increase in the hydropower generation in 85 % of the scenarios used, with the Salg being preferable in all the scenarios used, by improvement the performance of the GAOM. The execution times of the GAOM using these two algorithms were compared during all the scenarios used as shown in Fig. 9. This comparison indicated that the execution times of the Salg were less than those of the Falg by about 26 min in each scenario used. This shortcut in the execution time using the Salg happened because of the difference in the numbers of generations which were used.

**Table 4:** The increments and decrements of hydropower as a percentage using the GAOM with the two algorithms compared with the observed hydropower generated

Years	Observed hydropower generated (GWh)	Percentage of increment/decrement using the Falg (%)	Percentage of increment/decrement using the Salg (%)	Percentage of increment/decrement using the Salg adding Ev. and Pr. (%)
1989–1990	2521	*0.11*	*1.83*	−***0.29***
1990–1991	1277	*33.26*	*36.25*	*29.93*
1991–1992	2705	*13.68*	*15.97*	*12.88*
1992–1993	3470	*2.76*	*3.09*	*1.20*
1993–1994	2561	−***3.46***	−***0.82***	−***3.68***
1994–1995	3710	*0.43*	*0.87*	−***1.34***
1995–1996	2516	*5.38*	*7.18*	*3.98*
1996–1997	2645	−***4.46***	−***1.93***	−***6.29***
1997–1998	2465	*5.05*	*5.32*	*2.86*
1998–1999	1189	*8.23*	*16.77*	*10.29*
1999–2000	1084	*15.60*	*24.17*	*16.67*
2000–2001	1571	−***3.99***	−***1.79***	−***7.01***
2001–2002	2524	*1.71*	*1.94*	−***0.88***
2002–2003	2517	*24.37*	*24.86*	*21.62*
2003–2004	2935	*0.37*	*0.81*	−***1.82***
2004–2005	2193	*5.88*	*7.49*	*3.32*
2005–2006	2284	*24.87*	*26.95*	*23.39*
2006–2007	1586	*56.56*	*57.42*	*52.29*
2007–2008	1076	*13.65*	*16.80*	*11.91*
2008–2009	1650	*1.35*	*5.11*	*0.39*

The italics indicate to the increments of hydropower as percentages, whilst the bold italics indicate to the decrements of hydropower as percentages, achieved using the GAOM compared with the observed hydropower generated

In the third phase, the GAOM was applied using the same historical data for Mosul reservoir by using the Salg, which represents the best, taking into consideration the volumes of Ev and Pr. The values of the objective function obtained by using the Salg with and without taking into consideration the volumes of Ev and Pr were compared as shown in Fig. 8. In addition, the increments and decrements of hydropower generation achieved as percentages by using the Salg with and without taking into consideration the volumes of Ev and Pr were compared as shown in Table 4. These comparisons showed the reduction in the values of the objective function in all the twenty scenarios taking into consideration the volumes of Ev and Pr. This occurred because of the continuation of Ev during all the months of the year, in contrast to the Pr which happens just during 8 months of the year with respect to the Mosul reservoir. In addition, the depths of Ev are significantly higher than the Pr as shown in Fig. 7. All of these reflected negatively on the storage volume and thus on the values of objective function. In addition, those comparisons showed that the Salg with taking into consideration the volumes of Ev and Pr achieved an increase in the annual hydropower generation in 65 % of the scenarios used compared with the observed. Spill values from the spillway were zeros during all the time periods of twenty scenarios used. In addition, the releases from the bottom outlets were zeros during the periods of scenarios used except the some months which were shown in Table 5.

**Table 5:** Releases obtained from bottom outlet, using the Salg taking into consideration the Ev and Pr

Years	Month in water year	Release from bottom outlets (MCM)
1991–1992	May	7.86
1992–1993	May	1580
	June	124.6
1993–1994	April	0.56
1994–1995	May	31.4
1996–1997	May	0.37
1997–1998	May	0.12
2001–2002	May	32.88
2002–2003	May	0.17
2005–2006	May	1.35
2006–2007	May	16.5

**Fig. 10:**
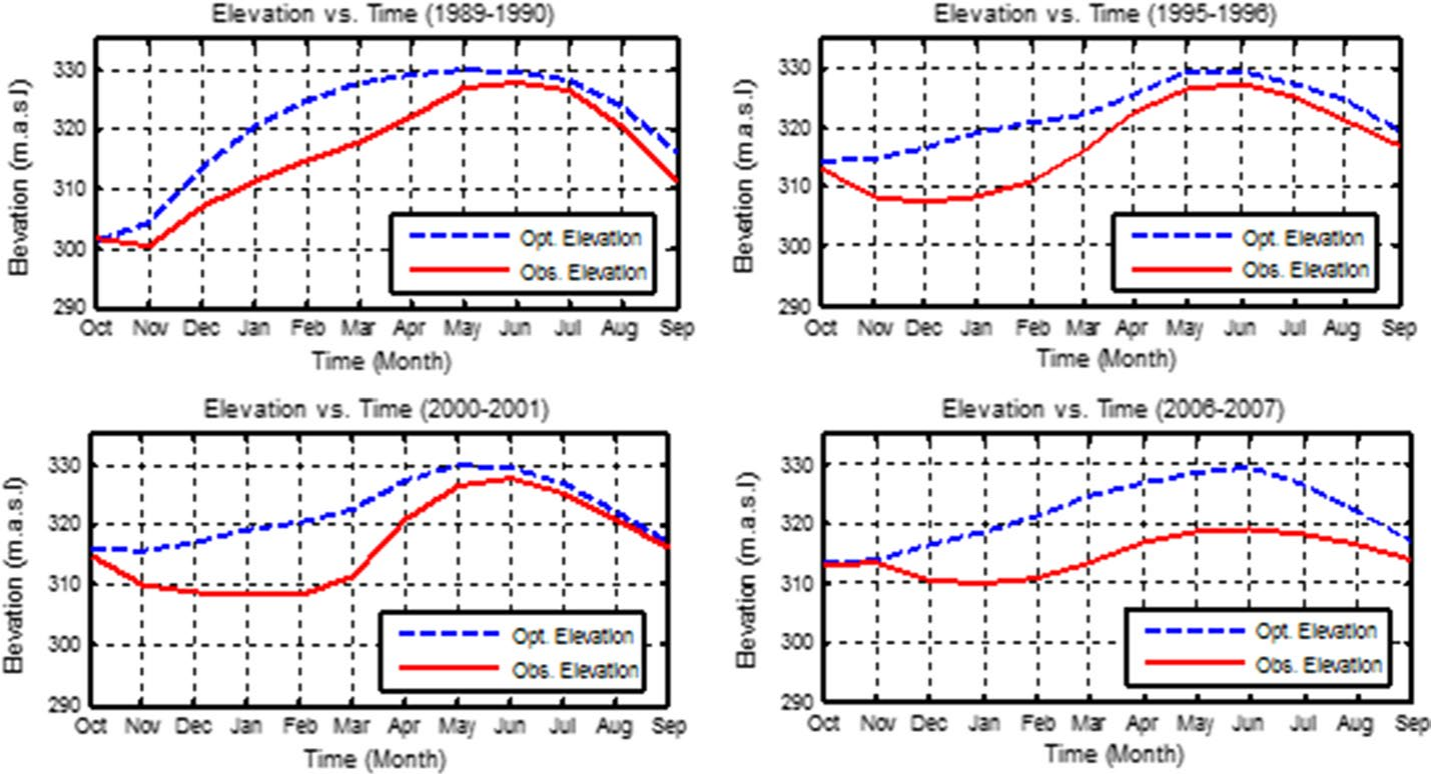
Observed elevations and optimal elevations using the Salg taking into consideration the Ev and Pr

**Fig. 11:**
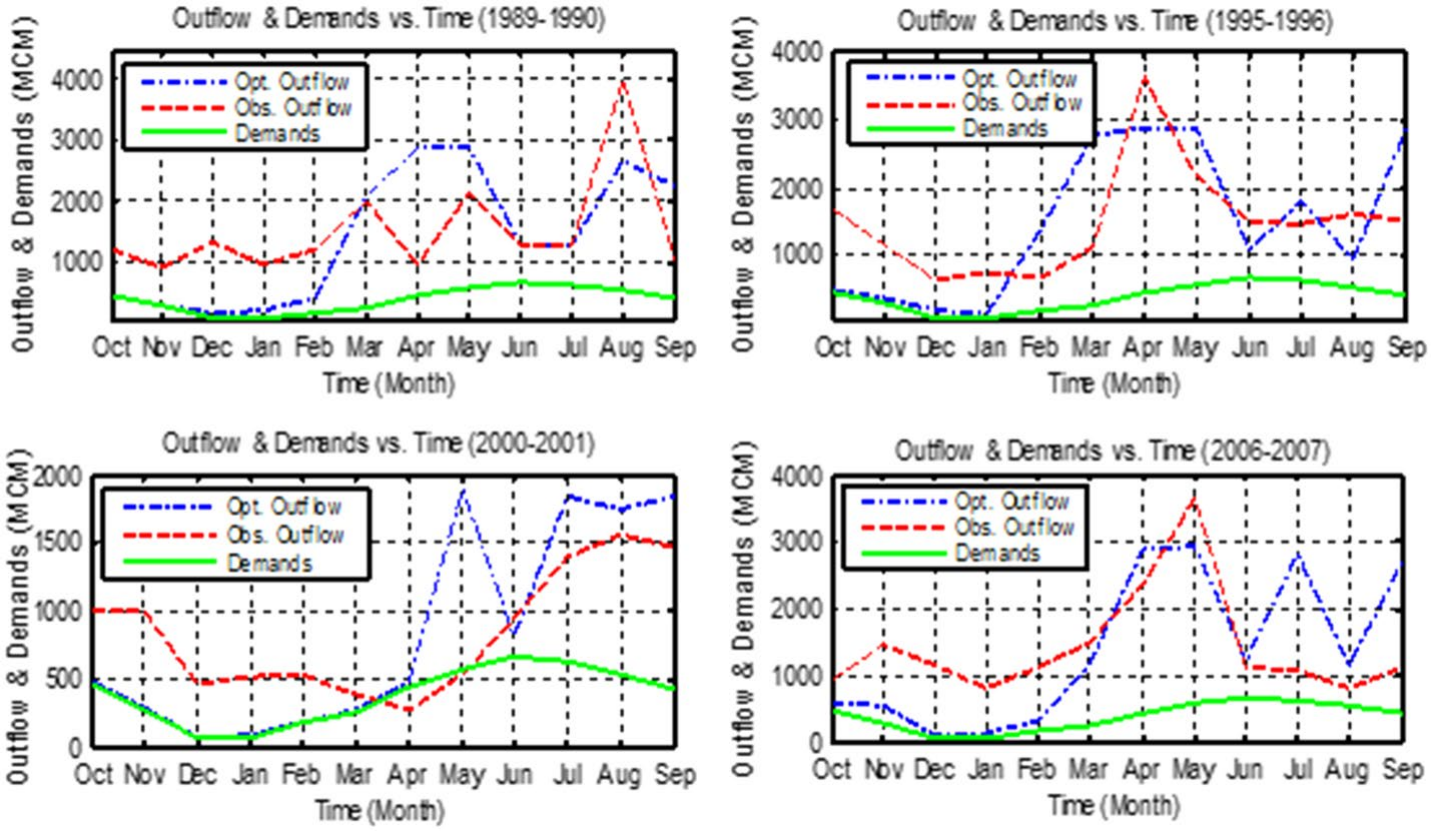
Observed outflows and optimal outflows using the Salg taking into consideration the Ev and Pr

A new rule curve was determined for each year by using the Salg as shown in Fig. 10 for 4 years, as examples. These new rules curves maintained good levels for storages during the majority of the 20 scenarios used, compared with the observed level. These new operation rules achieved all the requirements downstream the reservoir and managed to increase the annual hydropower production. Figure 11 shows the observed releases compared with the optimal releases for 4 years, as examples. This figure shows that optimal releases were not reduced to less than the monthly requirements.

## Conclusions

First, the performances of the two algorithms formulated in the GAOM, were evaluated without taking into consideration the volumes of Ev and Pr in the continuity equation. The Falg is based on the traditional simulation process of reservoir operation, whilst the Salg has enhanced the work of GAOM. Second, the GAOM was evaluated using the Salg with taking into consideration the effect of Ev and Pr.

The first and second phases of the results and discussion section, showed that the Salg is superior, where it achieved high values for the objective function in a short execution time compared with the Falg. The Salg was able to enhance the performance of GAOM through improve the traditional simulation process of reservoir operation by changing the population values of GA (releases to the powerhouse (*RP*_*t*_)) to increase the maximization of annual hydropower generation. From all of the previous results obtained using various scenarios, arguably that using the Salg in GAOM is the most capable to determine the optimal operating policy for the Mosul reservoir or any similar reservoir by increasing the optimal annual hydropower generation.

The third phase of the results and discussion section, showed that it is necessary inclusion the parameters of Ev and Pr in the continuity equation when using or creating a model simulates the dynamic processes of water storage system, such as the optimization and simulation models.

In addition, the GAOM maintained good levels of storage in the most of the scenarios used compared to the observed levels, which could be useful for recreation, especially in the summer. As well, the GAOM lost small amounts of water through only the bottom outlets during a few periods of operation.

In future, in order to apply this optimization model using future predictions, the model should be operated in conjunction with good stochastically generated data. In addition this GAOM can be developed to represent a single- or multi-reservoir system by using three modes of annual inflows (minimum, average, and maximum) during historical and synthetic inflows in order to determine the optimal policies.
